# Метастатическая феохромоцитома при синдроме множественной эндокринной неоплазии 2А типа

**DOI:** 10.14341/probl13332

**Published:** 2024-02-26

**Authors:** Д. В. Реброва, В. Ф. Русаков, Л. М. Краснов, Е. А. Федоров, И. К. Чинчук, Н. В. Ворохобина, Ш. Ш. Шихмагомедов, А. А. Семенов, Р. А. Черников, И. В. Слепцов, Г. И. Гафтон, Е. Н. Имянитов

**Affiliations:** Санкт-Петербургский государственный университет; Санкт-Петербургский государственный университет; Санкт-Петербургский государственный университет; Санкт-Петербургский государственный университет; Санкт-Петербургский государственный университет; Северо-Западный государственный медицинский университет им. И.И. Мечникова; Санкт-Петербургский государственный университет; Санкт-Петербургский государственный университет; Санкт-Петербургский государственный университет; Санкт-Петербургский государственный университет; Национальный медицинский исследовательский центр онкологии им. Н.Н. Петрова; Национальный медицинский исследовательский центр онкологии им. Н.Н. Петрова

**Keywords:** феохромоцитома, синдром множественной эндокринной неоплазии 2 типа, метастатическая феохромоцитома, злокачественная феохромоцитома, синдром МЭН2А, синдром МЭН

## Abstract

Феохромоцитома (Фхц) — редкая нейроэндокринная опухоль, исходящая из хромаффинных клеток мозгового вещества надпочечников. Эмбриологически, морфологически и функционально Фхц является симпатической надпочечниковой параганглиомой. По современным представлениям, Фхц относится к нейроэндокринным злокачественным новообразованиям в связи с возможным непредсказуемым проявлением агрессивного роста c метастазированием и инвазией в соседние органы и сосуды. До 25% всех случаев Фхц — это семейные формы. Одной из наиболее часто встречающихся семейных форм Фхц является синдром множественной эндокринной неоплазии 2А типа (синдром Сиппла, МЭН2А). При синдроме МЭН2А Фхц встречается с пенетрантностью до 50% — как одно- так и двусторонние, однако метастазы встречаются крайне редко. В подавляющем большинстве случаев при синдроме МЭН2А первичным источником отдаленных метастазов является медуллярная карцинома щитовидной железы (МКЩЖ), что затрудняет дифференциальную диагностику и поиск первичной опухоли.Мы представляем описание пациентки 40 лет с синдромом МЭН2А (мутация RET-протоонкогена p.Cys634Tyr) с дважды проведенным оперативным лечением МКЩЖ в анамнезе, с ежедневными симпато-адреналовыми кризами и критической артериальной гипертензией, с гигантской двусторонней Фхц (до 200 мм справа и до 150 мм слева), а также с синхронным крупным метастазом Фхц (до 50 мм) в лонную кость с деструкцией последней. Кроме того, диагностирован первичный гиперпаратиреоз. Пациентке поэтапно выполнены двусторонняя адреналэктомия, затем двусторонняя ревизия шеи, удаление правой верхней и правой нижней околощитовидных желез, остаточной ткани щитовидной железы, далее — резекция правой лонной кости с опухолью.

## Abstract (en)

Pheochromocytoma (PHEO) currently is considered to be malignant due to metastatic potential. One of the most common familial forms of PHEO is multiple endocrine neoplasia syndrome (MEN) type 2. The penetrance of PHEO in MEN2 syndrome is up to 50% of cases. It may be one- or two-sided, but metastases occur extremely rare. The fact that in majority of cases of MEN2 syndrome the source of distant metastases is medullary thyroid carcinoma (MTC) complicates differential diagnosis in case of PHEO metastasis.Isolated cases of PHEO with metastases to the lymph nodes, lungs, liver, bones, brain in MEN2 patients were described. In the available literature, we have found a description of 31 cases of metastatic PHEO in MEN2 syndrome. The available data of those cases is presented as a table in the article.We present a description of a 40-year-old woman with MEN2A syndrome (mutation of the RET proto-oncogene p.Cys634Tyr), with a history of twice-performed surgical treatment of MTC, with daily crises of arterial hypertension accompanied by vegetative symptoms, with a giant bilateral PHEO (up to 200 m on the right and up to 150 mm on the left) with synchronous large metastasis (up to 50 mm) into the pubic bone with the destruction. The patient underwent several surgeries: bilateral adrenalectomy, then a bilateral revision of the neck, removal of the right upper and right lower parathyroid glands, residual thyroid tissue, then resection of the right pubic bone with a tumor.

## АКТУАЛЬНОСТЬ

Феохромоцитома/параганглиома (Фхц/ПГ) — нейроэндокринная опухоль из хромаффиной ткани мозгового вещества надпочечника. Фхц является частным случаем симпатической надпочечниковой ПГ. С 2017 г., в соответствии с классификацией опухолей эндокринных органов ВОЗ 4-го пересмотра, Фхц/ПГ отнесены к злокачественным новообразованиям, в связи с чем термины «злокачественная» и «доброкачественная» заменены на «метастатическую» и «неметастатическую» [1].

Метастазы Фхц/ПГ определяются как депозиты, выявляемые в локализациях, где в норме не встречается хромаффинная ткань [2]. Принято считать, что около 10% Фхц и около 40% симпатических ПГ обладают метастатическим потенциалом [3].

По современным представлениям, до 50% Фхц/ПГ ассоциированы с наличием генетических мутаций, как герминальных, так и соматических [4]. Одной из наиболее часто встречающихся семейных форм заболевания является синдром множественной эндокринной неоплазии 2 А типа (синдром Сиппла, МЭН2А). При синдроме МЭН2А Фхц встречается с различной пенетрантностью в зависимости от экзона, в котором произошла мутация, и достигает 50% случаев семейных форм. Наблюдаются как односторонние, так и двусторонние опухоли мозговой ткани надпочечников, однако вненадпочечниковая локализация и появление метастазов не характерно для данной группы пациентов [5]. При синдроме МЭН2А пенетрантность развития медуллярной карциномы щитовидной железы (МКЩЖ) составляет практически 100%, при этом нередко наблюдается метастазирование, в связи с чем выявленные отдаленные метастазы Фхц у данной категории пациентов могут ошибочно расцениваться как метастазы МКЩЖ, что может существенно повлиять на выбор правильной диагностической и лечебной тактики.

В литературе описаны единичные случаи Фхц при МЭН2 с наличием отдаленных метастазов в лимфатические узлы, легкие, печень, кости, головной мозг. Мы представляем описание пациентки с синдромом МЭН2А с двусторонней Фхц с крупным метастазом в лонную кость, а также обзор литературы подобных редких клинических случаев.

## ОПИСАНИЕ СЛУЧАЯ

Пациентка К., 40 лет, поступила в Клинику высоких медицинских технологий им. Н.И. Пирогова Санкт-Петербургского государственного университета (КВМТ СПбГУ) с жалобами на приступообразное повышение артериального давления (АД) максимально до 210/120 мм рт.ст., общую слабость, утомляемость. Приступы сопровождались выраженным сердцебиением, ощущением жара, профузной потливостью, чувством тревоги и страха, побледнением кончиков пальцев рук. Подобные эпизоды с различной степенью выраженности вегетативных и неврологичексих проявлений возникали 1–2 раза в день, длились 5–15 минут, купировались самостоятельно с последующей выраженной общей слабостью, снижением уровня АД до 90–100/60 мм рт.ст. В межприступный период показатели АД — в пределах нормальных значений: 110–120/70–80 мм рт.ст. без гипотензивной терапии.

Из анамнеза известно, что в 1993 г. пациентке выполнена гемитиреоидэктомия справа по поводу узлового нетоксического зоба. В 2009 г. при ультразвуковом исследовании (УЗИ) выявлен узел в левой доле ЩЖ, прооперирована в объеме левосторонней гемитиреоидэктомии с удалением паратрахеальной клетчатки слева. Выписные справки и гистологические заключения операций в 1993 и 2009 гг. утеряны, под наблюдением онколога не состояла С 2009 г. принимает левотироксин в заместительной дозе 100 мкг в сутки. В 2010 г. при плановом УЗИ шеи выявлен увеличенный лимфатический узел слева, проведена левосторонняя лимфаденэктомия. Гистологическое заключение: вторичный лимфаденит медуллярного строения. Несмотря на молодой возраст дебюта МКЩЖ у пациентки, наличие семейных форм заболевания не предполагалось и обследование на ее возможность не проводилось.

В течение года до поступления в КВМТ, появились эпизоды кризового повышения артериального давления до 210 и 120 мм рт.ст. с выраженной вегетативной симптоматикой, в связи с чем и обратилась к терапевту по месту жительства. В рамках комплексного обследования по поводу артериальной гипертензии было выполнено УЗИ органов брюшной полости, при котором выявлены образования обоих надпочечников крупных размеров. При компьютерной томографии (КТ) органов брюшной полости в области обоих надпочечников визуализированы крупные многоузловые солидные образования с гетерогенной структурой, размером до 121х115х210 мм справа и 117х97х159 мм слева, сдавливающие и оттесняющие кпереди печень, без признаков инвазии в паренхиму последней. В правой лонной кости на уровне лонного сочленения выделен остеолитический очаг с небольшим параоссальным компонентом размером до 38 мм.

Проведена позитронно-эмиссионная томография, совмещенная с КТ (ПЭТ–КТ) от затылочной кости до средней трети бедра с 18-фтордезоксиглюкозой (18-ФДГ). В проекции обоих надпочечников определены неоднородные образования с участками повышенной и пониженной плотности и мелкими единичными кальцинатами, с повышенной метаболической активностью ФГД (SUVmax=5,5), размерами до 98 мм, смещающие все соседние органы. В лобковой кости справа определяется образование размерами 31х35 мм, с повышенной метаболической активностью ФДГ (SUVmax=6,2), вызывающее частичную деструкцию кости. Заключение: ПЭТ–КТ-картина объемных образований забрюшинного пространства специфического характера, метастатическое поражение правой лобковой кости с повышенной метаболической активностью ФДГ.

Амбулаторно по месту жительства выполнена трепан-биопсия образования надпочечника с проведением иммуногистохимического исследования (ИГХ). Микроскопически: материал крайне скудный, представлен точечными фрагментами опухоли солидно-трабекулярного строения из полиморфных округло-овальных клеток крупных и средних размеров. Опухолевые клетки экспрессируют виметин, CD56, хромогранин А, синаптофизин; единичные мелкие клетки экспрессируют протеин S100. Индекс пролиферативной активности Ki67 — 3–5%. Заключение: иммуноморфологическая картина наиболее соответствует феохромоцитоме; с учетом наличия вторичных очагов опухоли — злокачественной.

При обследовании кортизол крови утром натощак повышен до 32,41 мкг/дл (6,2–19,4), кальцитонин — 9,4 пмоль/л (до 5).

С целью дообследования и уточнения дальнейшей тактики ведения направлена в КВМТ СПбГУ.

При поступлении состояние пациентки удовлетворительное. Рост — 169 см, вес — 57 кг, индекс массы тела (ИМТ) — 19,8 кг/м², температура тела — 36,7 ˚С. Кожные покровы чистые, естественной окраски, обычной влажности, отсутствуют ганглионевромы, лихеноидный амилоидоз на коже. Видимые слизистые оболочки чистые, обычной окраски. Щитовидная железа не пальпируется, послеоперационные рубцы на передней поверхности шеи. Регионарные и периферические лимфоузлы не увеличены. Тоны сердца ясные, ритмичные. Артериальное давление — 140/90 мм рт.ст., пульс — 88 уд/мин, ритмичный. Число дыханий — 18 в 1 мин. Дыхание везикулярное, хрипов нет. При поверхностной пальпации живот мягкий, безболезненный во всех отделах. Глубокая пальпация живота не проводилась в связи с угрозой провоцирования симпатоадреналового криза. Физиологические отправления в норме. Отеков нет.

При лабораторном обследовании в клиническом анализе крови патологических отклонений выявлено не было, скорость оседания эритроцитов (СОЭ) ускорена до 26 мм/ч (1–20). В биохимическом анализе крови отмечалось повышение уровня гамма-глутамилтрансферазы (ГГТ) до 42,8 Е/л (до 38). АСТ, АЛТ, билирубин, креатинин находились в пределах референсных значений. В коагулограмме отмечено небольшое повышение международного нормализованного отношения (МНО) до 1,22 (0,8–1,2), значимое повышение уровня фибриногена до 7,1 г/л (2–4), остальные показатели без патологических изменений. В общем анализе мочи отклонений от нормы не было.

При исследовании фосфорно-кальциевого обмена в крови обнаружено повышение паратгормона до 13,1 пмоль/л (1,3–9,3) в сочетании с повышенным ионизированным кальцием до 1,38 ммоль/л (1,13–1,31). Адренокортикотропный гормон (АКТГ) был 2,487 пмоль/л (1,034–10,736), базальный кортизол — 421,5 нмоль/л (185–624), альдостерон — 38,9 пг/мл (18,8–256,7), прямой ренин — 74,29 мкМЕ/мл (2,8–39,9), альдостерон-рениновое соотношение (АРС) — 0,52, дегидроэпиандростерон (ДЭА)-сульфат — 0,5 мкмоль/л (0,62–7,22), тиреотропный гормон (ТТГ) — 7,442 мкМЕ/мл (0,4–4,0). Определено значимое повышение уровней фракционированных метанефринов плазмы: метанефрин >3600 пг/мл (до 65), норметанефрин >7200 пг/мл (до 196). В суточной моче кортизол составил 550,25 нмоль/сут (160–1112), экскреция общих метанефринов с мочой (LS-MS) была >600 мкг/сут (до 350). На фоне ночного супрессивного теста с 1 мг дексаметазона кортизол — 239,95 нмоль/л (отсутствие супрессии, по-видимому, вследствие гиперстимуляции гипоталомо-гипофизарно-надпочечниковой системы гиперконцентрацией катехоламинов). Уровень кальцитонина был повышен и составил 15,7 пг/мл (норма для некурящих до 4,8).

При исследовании маркеров нейроэндокринных опухолей в крови раковый эмбриональный антиген (РЭА) находился в пределах референсных значений и составил 1,15 нг/мл (норма для некурящих до 3), но были повышены уровни хромогранина А до 3182,4 мкг/л (до 125) и нейроспецифической энолазы (NSE) до 27,39 мкг/л (до 18,3).

При электрокардиографии (ЭКГ): синусовый ритм с частотой сердечных сокращений (ЧСС) 83 уд/мин, умеренное нарушение реполяризации, характерное для ЭКГ-признаков гипертрофии левого желудочка (ЛЖ) и диффузных неспецифических изменения миокарда ЛЖ.

По данным эхокардиографии: полости сердца не расширены, миокард ЛЖ утолщен (масса миокарда 167 г), индекс массы миокарда повышен до 101,8 г/м², отмечался гиперкинез миокарда желудочков с ускорением кровотока на всех клапанах сердца, гипертрофия миокарда ЛЖ.

Гиперкальциемия, гиперпаратиремия у пациентки МКЩЖ и новообразованием обоих надпочечников с чрезвычайно высоким содержанием метанефринов стали основанием заподозрить синдром МЭН2А.

При повторной КТ органов шеи, грудной клетки, брюшной полости и малого таза с болюсным контрастным усилением: КТ-признаков патологических образований органов грудной клетки и шеи не выявлено, визуализируется участок разрежения костной ткани рукоятки грудины, без четких контуров, патологических образований переднего средостения не выявлено, желчный пузырь в размерах не увеличен, в просвете — рентгенконтрастные конкременты до 6 мм в диаметре и плотностью +700 HU. Левый надпочечник увеличен за счет многоузлового образования размерами 110х90х150 мм, плотностью +30 HU. В I фазу накопление контрастного вещества до +60 HU, во II фазу — до +60 HU, через 10 минут плотность образования +50 HU. Правый надпочечник увеличен за счет многоузлового образования размерами 120х120х200 мм, плотностью +30 HU. В I фазу накопление контрастного вещества — до +80 HU, во II фазу — до +75 HU, через 10 минут плотность образования +50 HU. Структура образований обоих надпочечников неоднородная за счет многочисленных зон некроза в центре. Образования обильно васкуляризированы. Надпочечниковые вены широкие, до 10 мм в диаметре (рис. 1). Данных за инвазию окружающих структур, сосудов не получено. Данных за тромбоз нижней полой вены не получено. Лимфатические узлы не увеличены. Очаг остеолитической деструкции в правой лонной кости с наличием мягкотканного компонента размерами 50х40х40 мм (рис. 2).

**Figure:**
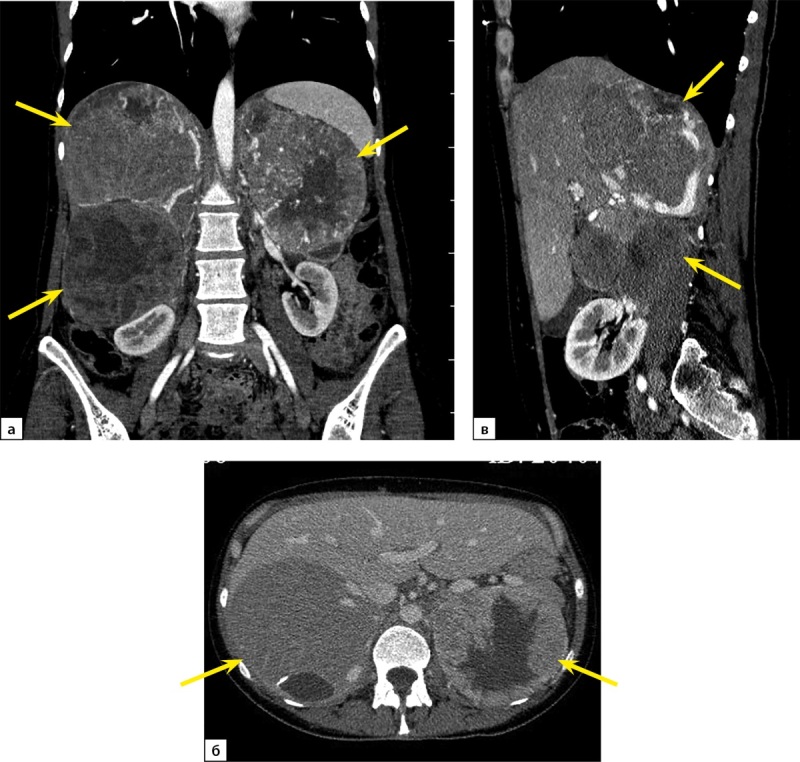
Рисунок 1. Компьютерная томография органов брюшной полости с контрастированием: а — артериальная фаза, коронарная плоскость; б — венозная фаза, аксиальная плоскость; в — артериальная фаза, сагиттальная плоскость. Стрелками отмечены гигантские множественные феохромоцитомы обоих надпочечников.

**Figure:**
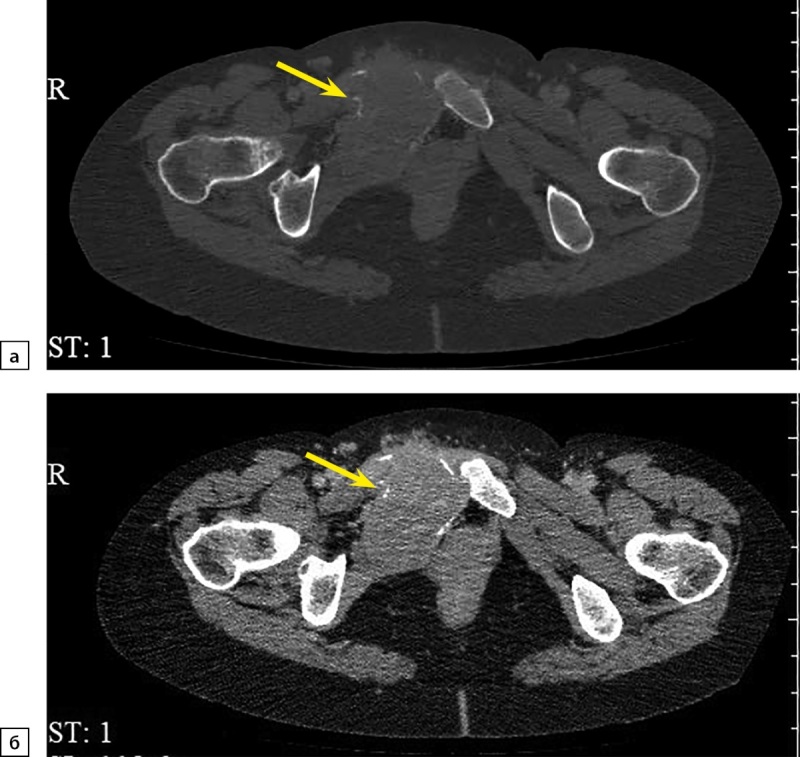
Рисунок 2. Компьютерная томография органов малого таза с контрастированием: а — артериальная фаза, костное окно, аксиальная плоскость; б — отсроченная фаза, мягкотканное окно, аксиальная плоскость. Стрелками отмечен метастаз феохромоцитомы в лонную кость.

Для визуализации околощитовидных желез проведены полипозиционная сцинтиграфия околощитовидных желез с Тс99-технетрилом и однофотонная эмиссионная КТ (ОФЭКТ) грудной клетки, по данным которых выделяется зона фиксации радиофармпрепарата (РФП) размером примерно 25х15 мм в проекции рукоятки грудины, левее срединной линии (паратиреоидная ткань?). Кроме того, отмечено повышенное накопление РФП в проекции подключичных лимфоузлов с обеих сторон. В проекции костей таза зон патологической фиксации РФП не выявлено.

По данным обследования установлен диагноз: «Синдром множественной эндокринной неоплазии 2А типа (синдром Сиппла), клинически. Феохромоцитома обоих надпочечников cT3NxM1 (os pub.), пароксизмальная форма. Симптоматическая артериальная гипертензия с частыми симпатоадреналовыми кризами. Дисметаболическая миокардиодистрофия. Хроническая сердечная недостаточность 2А стадии, II функциональный класс по классификации Нью-Йоркской ассоциации сердца. Медуллярная карцинома щитовидной железы pT4aN1bM0, ст.IVа. Гемитиреоидэктомия от 1993 г., экстирпация культи щитовидной железы с удалением паратрахеальной клетчатки от 2009 г., шейная лимфаденэктомия слева от 2010 г. Первичный гиперпаратиреоз, бессимптомная форма. Гиперкальциемия легкой степени.

Желчнокаменная болезнь. Хронический калькулезный холецистит, вне обострения».

В стационаре проводилась стандартная для пациентов с Фхц подготовка альфа1-адреноблокатором (доксазозин) с постепенным повышением дозировки до 8 мг в сутки. На этом фоне достигнуто прекращение симпато-адреналовых приступов, показатели АД не превышало 150/100 мм рт.ст., пульс — 62–78 удара в минуту. Бета-адреноблокаторы в процессе предоперационной подготовки не назначались.

Пациентке выполнена двусторонняя адреналэктомия открытым доступом с удалением лимфатических узлов брыжейки и парааортальной клетчатки. Из отдельного разреза на уровне лонного сочленения выполнена биопсия костного дефекта лонной кости, сделан мазок детрита. На фоне мобилизации как левого, так и правого надпочечников отмечался подъем уровня АД до 210–230/100–110 мм рт.ст., частоты сердечных сокращений (ЧСС) до 98 ударов в минуту, что корректировалось введением нитроглицерина и эсмолола. После адреналэктомии слева и справа отмечалось резкое транзиторное снижение уровня АД до 63–55/38–42 мм рт.ст., что корректировалось введением инфузионных сред, дофамина, адреналина и норадреналина.

По данным гистологического заключения операционного материала: феохромоцитома правого и левого надпочечников (20 см и 15 см), с очагами некроза и сосудистой инвазии; в лимфатических узлах брыжейки и парааортальной клетчатки без метастатического поражения, умеренная лимфофолликулярная гиперплазия, синусовый гистиоцитоз. ИГХ: опухолевые клетки интенсивно экспрессируют Chromogranin А, Synaptophysin; сустентокулярные клетки визуализируются S100 протеином, распределены неравномерно, выявляются обширные сливающиеся поля без наличия сустентоцитов; экспрессия р53 в 1%, р21 в 1–5%; индекс пролиферативной активности Ki67 1–5%.

Цитологическое заключение биоптата дефекта лонной кости: в материале обилие крупных мономорфных клеток с зернистой цитоплазмой и с крупными подчеркнуто округло-овальными гиперхромными ядрами, с умеренным клеточно-ядерным полиморфизмом, что соответствует метастазу солидной крупноклеточной опухоли с нейроэндокринной дифференцировкой. ИГХ из парафинового блока препарата трепан-биопсии: опухолевые клетки интенсивно экспрессируют Chromogranin А, Synaptophysin, не экспрессируют S100 протеин, Calcitonin, TTF1. Индекс пролиферативной активности Ki67 составил 7–10%. Цитологическая картина и иммунофенотип опухоли лонной кости соответствуют метастазу феохромоцитомы.

Послеоперационный период протекал гладко, без осложнений. При контроле АД в течение двух суток после операции отмечалась умеренная гипертензия до 145 и 90 мм рт.ст. с последующей нормализацией АД. Выписана на 9-е сутки для амбулаторного лечения под наблюдением онколога, хирурга, эндокринолога с рекомендацией постоянного приема левотироксина 125 мкг в сутки, доксазозина 4 мг, гидрокортизона 45 мг в сутки, флудрокортизона 0,1 мг в сутки, алендроната 70 мг в неделю. Прием доксазозина в уменьшенной дозировке под контролем АД решено сохранить в связи с возможной гиперсекрецией катехоламинов костным метастазом Фхц.

По данным молекулярно-генетического исследования, выявлена мутация RET-протоонкогена c.1901G>A (p.Cys634Tyr). У пациентки двое детей, рекомендовано их генетическое типирование на носительство выявленной мутации.

Через 2 месяца осуществлена повторная госпитализирована в КВМТ СПбГУ с целью оперативного лечения по поводу первичного гиперпаратиреоза.

При поступлении состояние удовлетворительное. На фоне проводимой заместительной и гипотензивной терапии показатели гемодинамики в пределах нормальных значений, вегетативные проявления отсутствуют. Паратгормон — 129 пг/мл (15–65), кальций ионизированный — 1,44 ммоль/л (1,13–1,31), кальцитонин — 5 пг/мл (до 5), метанефрин — 15,1 пг/мл (до 65), норметанефрин — 2412,9 пг/мл (до 196), хромогранин А — 654,7 мкг/л (до 125). Экскреция с суточной мочой общих метанефринов составила 5,6 мкг/сут (до 350).

Повышение уровня норметанефрина расценено как результат гиперпродукции костным метастазом Фхц норадреналина. Обращает внимание нормализация концентрации кальцитонина, что можно объяснить паранеопластической секрецией кальцитонина удаленными феохромоцитомами.

При КТ шеи с контрастированием вдоль передней поверхности щитовидного хряща, слева от срединной линии, выявлено образование 15х9х6 мм, интенсивно накапливающее контрастное вещество, увеличенных и структурно измененных лимфатических узлов не обнаружено, трахея не деформирована.

Пациентке выполнена двусторонняя ревизия шеи, удаление правой верхней и правой нижней околощитовидных желез, остаточной ткани щитовидной железы, имплантация участка околощитовидной железы в musculus brachioradialis. По результатам срочного интраоперационного гистологического исследования во всех фрагментах определены множественные микрофолликулярные и ацинарные комплексы мономорфных эпителиальных клеток, местами с признаками онкоцитарной дифференцировки, соответствующие ткани околощитовидных желез. Был проведен интраоперационный контроль уровня паратгормона: проба 1 (прединцизионная) — 16,4 пмоль/л (1,3–9,3), проба 2 (предэксцизионная) —15,2 пмоль/л, проба 3 (через 10 минут после удаления) — 3,4 пмоль/л. При гистологическом исследовании подтверждено наличие аденомы правой верхней околощитовидной железы (0,7 см), фрагмента ткани правой нижней околощитовидной железы с признаками гиперплазии (0,8 см), нормофолликулярного строения.

Послеоперационный период протекал гладко, без осложнений. Достигнута нормокальциемия.

При контроле КТ органов малого таза с контрастированием отмечен рост патологического образования, вызывающего деструкцию верхней и нижней ветвей правой лонной кости, размером 80х57х55 мм (ранее 50х40х40 мм) с пролабацией в полость таза. Выявлена деформация правой стенки мочевого пузыря, прилежащая жировая клетчатка не инфильтрирована.

Пациентка выписана из стационара. На момент выписки рекомендован прием доксазозина 4 мг в сутки, гидрокортизона 35 мг в сутки, карбоната кальция 1000 мг в сутки, альфакальцидола 1 мкг в сутки.

Через 2 месяца в НМИЦ онкологии им. Н.Н. Петрова выполнена резекция правой лонной кости с опухолью. По результатам гистологического исследования подтверждено, что опухоль является метастазом феохромоцитомы.

Через 1 месяц после выписки при телефонном контакте с пациенткой она сообщила об улучшении общего самочувствия. Еще через 1 месяц состоялся контакт по электронной почте: состояние стабильное, было рекомендовано контрольное обследование через 1 месяц. Дальнейший контакт с пациенткой был утерян.

## ОБСУЖДЕНИЕ

Метастатическая Фхц в рамках синдрома МЭН 2 типа встречается крайне редко. По результатам ретроспективного анализа историй 272 пациентов с метастатической Фхц/ПГ с 1960 по 2016 гг. в исследовании Hamidi O. и соавт. (2017 г.) из клиники Мейо США только у 2 больных был диагностирован синдром МЭН2А, тогда как синдром МЭН 2В типа не был выявлен ни в одном из случаев [6]. В работе Thosani S. и соавт. (2013 г.) из 319 пациентов с синдромом МЭН 2 типа только у 85 была выявлена Фхц, при этом в процессе динамического наблюдения ни одного случая метастатического поражения установлено не было [7]. Схожие данные получены Oishi S. и соавт. (1990 г.) в оценке 90 случаев синдрома МЭН 2 типа в Японии [8]. В исследовании Kotecka-Blicharz A. и соавт. (2016 г.) из 228 RET-положительных пациентов Фхц была выявлена у 18% из них, 1 случай с метастазами в лимфатические узлы [9]. По данным анализа Kumar S. и соавт. (2021 г.) базы 450 случаев Фхц/ПГ, у 23 пациентов (5,1%) был установлен синдром МЭН 2 типа (19 — с МЭН 2А, 4 — с МЭН 2В), у 2 пациентов с МЭН 2А типа была выявлена метастатическая Фхц [10]. В ретроспективном одноцентровом исследовании Rajan S. и соавт. (2016 г.) историй 208 пациентов с Фхц синдром МЭН 2 типа диагностирован у 24 больных, при этом не было выявлено ни одной метастатической Фхц в группе пациентов с МЭН 2 типа по сравнению с 7 случаями из132 (5,3%) спорадических форм [11]. По результатам метаанализа литературы 3063 пациентов с Фхц/ПГ распространенность метастатических форм, ассоциированных с синдромом МЭН 2 типа, составила всего 0,05% [10].

В доступной литературе найдено описание 31 случая МЭН 2 типа с метастатической Фхц, подобных представленному нами, информация о которых обобщена в таблице 1. Кроме того, имеется случай успешного лечения 21-летней пациентки с МЭН 2А типа с инвазивным ростом Фхц с распространением опухолевого тромба в нижнюю полую вену и правое предсердие, без метастазов [29][34].

**Table:** Таблица 1. Клинические случаи метастатической феохромоцитомы (ФЕО) при синдроме множественной эндокринной неоплазии (МЭН) 2 типа * — сестры с семейным анамнезом метастатической ФЕО в рамках МЭН2А;** — сочетание метастазов МКЩЖ и ФЕО в печень, установлено по биопсии с ИГХ;*** — сочетание с метастазами рака предстательной железы, верификация по аутопсии по результатам гистологии с ИГХ.

№ п/п	Публикация	Пол	Возраст	Тип МЭН	Мутация в кодоне RET	Латерализация ФЕО	Максимальный размер первичной опухоли, см	Оперативное удаление первичной опухоли	Локализация метастазов	Смерть от заболевания
1	Carney J.A. и соавт. (1976) [12]	Ж	29	2А		Двустор.	13	+		+
2		Ж	23	2В		Двустор.		+		+
3		Ж*	28	2А		Двустор.	12	+		
4		Ж*	18	2А		Двустор.	5	+		
5	Wilson R.A., Ibanez M.L. (1978) [13]	Ж	53	2А		Двустор.	12	+	Печень	- (умерла от прободной язвы желудка)
6		Ж	38	2А		Двустор.	15	+	Печень	- (умерла от РМЖ)
7	Westfried M. и соавт. (1978) [14]	М	49	2			19		Легкие, перикард	
8	Sissin J.C. и соавт. (1984) [15]	Ж	26	2А		Двустор.	11		Печень	
9	Spapen H. и соавт. (1989) [16]	Ж	37	2А		Двустор.		+	Легкие	
10	Oishi S. и соавт. (1990) [8]	Ж	40			Двустор.				+
11		М	26			Левостор.				
12	Namba H. и соавт. (1992) [17]	М	33	2А		Левостор.			Легкие, печень	
13	Bonnin F. и соавт. (1994) [18]	М	35	2А		Двустор.	6	+	Лимфатические узлы	
14	Sasaki M. и соавт. (1994) [19]	Ж	39	2А		Двустор.			Легкие, печень	+
15	Scopsi L. и соавт. (1996) [20]	М	28	2В		Двустор.	12	+	Кости	
16	Hinze R. и соавт. (2000) [21]	М**	53	2А	634	Двустор.	9,4	+	Печень	
17	Gentle S. и соавт. (2001) [22]	Ж	31	2А		Левостор.		+	Головной мозг	
18	Hamdan A. и соавт. (2002) [23]	М	47	2А	634	Двустор.	7	+	Кости, печень	
19	Ishida E. и соавт. (2004) [24]	М***	65	2А		Двустор.	8	-	Печень	+
20	Gullu S. и соавт. (2005) [25]	Ж	34	2А	634	Двустор.		-	Поджелудочная железа	
21	Duquia R.P. и соавт. (2006) [26]	М	41	2В		Двустор.		+	Кожа	+ (послеоперационный сепсис)
22	Szalat A. и соавт. (2011) [27]	Ж	24	2В		Двустор.		+		+
23	Crona J. и соавт. (2014) [28]	М	65	2А	804	Одностор.	9,5	+		
24	Lang B.H.H. и соавт. (2015) [29]	Ж	47	2А	634	Правостор.	10	+	Кости	-
25	Martins A.F. и соавт. (2016) [30]	Ж	55	2А	531	Двустор.			Печень, паравертебрально	-
26	Kotecka-Blicharz A. и соавт. (2016) [9]	Ж	39	2	791	Одностор.	10	+	Лимфатические узлы	-
27	Pal R. и соавт. (2018) [31]	Ж	45	2А	634	Двустор.		+	По брюшине, под почками, в печени	
28	Ma X. и соавт. (2020) [32]	Ж	25	2А	634	Двустор.	8	+		
29	Jester G. и соавт. (2021) [33]	М	19	2В	918	Правостор.	11		Кости, легкие	+
30	Kumar S. и соавт., (2021) [10]	Ж	54	2А	618	Правостор.	6,1		Кости	-
31		Ж	45	2А	634	Двустор.	8,3		Печень	+
32	Описанный случай	Ж	40	2А	634	Двустор.	20	+	Кости	

## ЗАКЛЮЧЕНИЕ

На современном этапе развития хирургической и анестезиологической службы при стандартной предоперационной подготовке смертность от осложнений Фхц/ПГ снизилась до единичных случаев. Однако наличие отдаленных метастазов Фхц/ПГ существенно ухудшает прогноз пациентов. Редкость метастатической Фхц при синдроме МЭН 2 типа, трудность дифференциальной диагностики источника метастазов (МКЩЖ или Фхц) у данной категории больных представляет практический интерес для врачей различных специальностей.

## ДОПОЛНИТЕЛЬНАЯ ИНФОРМАЦИЯ

Источники финансирования. Работа выполнена по инициативе авторов без привлечения финансирования.

Конфликт интересов. Авторы декларируют отсутствие явных и потенциальных конфликтов интересов, связанных с содержанием настоящей статьи.

Участие авторов. Все авторы одобрили финальную версию статьи перед публикацией, выразили согласие нести ответственность за все аспекты работы, подразумевающую надлежащее изучение и решение вопросов, связанных с точностью или добросовестностью любой части работы.
